# Barley Genes as Tools to Confer Abiotic Stress Tolerance in Crops

**DOI:** 10.3389/fpls.2016.01137

**Published:** 2016-08-03

**Authors:** Filiz Gürel, Zahide N. Öztürk, Cüneyt Uçarlı, Daniele Rosellini

**Affiliations:** ^1^Department of Molecular Biology and Genetics, Faculty of Science, Istanbul UniversityIstanbul, Turkey; ^2^Department of Agricultural Genetic Engineering, Ayhan Şahenk Faculty of Agricultural Sciences and Technologies, Niğde UniversityNiğde, Turkey; ^3^Department of Agricultural, Food, and Environmental Sciences, University of PerugiaPerugia, Italy

**Keywords:** drought, *Hordeum vulgare*, LEA proteins, salinity, transcription factors

## Abstract

Barley is one of the oldest cultivated crops in the world with a high adaptive capacity. The natural tolerance of barley to stress has led to increasing interest in identification of stress responsive genes through small/large-scale omics studies, comparative genomics, and overexpression of some of these genes by genetic transformation. Two major categories of proteins involved in stress tolerance are transcription factors (TFs) responsible from the re-programming of the metabolism in stress environment, and genes encoding Late Embryogenesis Abundant (LEA) proteins, antioxidant enzymes, osmolytes, and transporters. Constitutive overexpression of several barley TFs, such as C-repeat binding factors (*HvCBF4*), dehydration-responsive element-binding factors *(HvDREB1)*, and WRKYs *(HvWRKY38)*, in transgenic plants resulted in higher tolerance to drought and salinity, possibly by effectively altering the expression levels of stress tolerance genes due to their higher DNA binding affinity. Na^+^/H^+^ antiporters, channel proteins, and lipid transporters can also be the strong candidates for engineering plants for tolerance to salinity and low temperatures.

## Introduction

Drought, salinity, high or low temperatures, frost, flooding, alkaline soil, and excess or deficiency of minerals like boron and aluminum can have significant adverse effects on agricultural production (Atkinson and Urwin, 2012). Particularly, drought and soil salinity even threat plant biodiversity in arid and semi-arid regions.

Plants have various protective mechanisms for coping with abiotic stress conditions. Both mechanisms based on single genes and complex regulatory pathways involved in stress tolerance and/or adaptation have been described in plants, and partially resolved by omics approaches of system biology (Gupta et al., 2013). Knowledge of the molecular basis of stress tolerance and adaptation is essential to develop crop cultivars with improved stress tolerance.

Barley (*Hordeum vulgare* L.) is one of the oldest cereal crops known to be cultivated since about 10,000 years in a region located between the Nile (Egypt) and Tigris Rivers (Iraq), also including Southern Turkey, Israel, Lebanon, Jordan, and Syria. It has a natural tolerance to drought, salinity, and fungal diseases, thus making it a model organism in stress biology research. Indeed, a barley plant was shown to complete its life-cycle before using all the available soil water, even in high salt concentrations and defined as the most salt-tolerant cereal (Munns et al., 2006).

Substantial work has been done to map genetic determinants controlling abiotic stress tolerance, which was the object of QTLs, meta-analysis that indicated the importance of 2H and 5H chromosomes (Li et al., 2013). In addition, molecular responses to drought, salinity, boron toxicity, cold acclimation, and high temperature have been revealed by high-throughput transcriptomic analyses (Ozturk et al., 2002; Svensson et al., 2006; Guo et al., 2009; Mangelsen et al., 2011; Tombuloglu et al., 2013; Bedada et al., 2014). Transcriptomic approaches have provided a large amount of data that enable researchers to identify major pathways and key proteins contributing to stress tolerance in barley. These major pathways are controlled by partially overlapping signaling components including abscisic acid (ABA), salicylic acid (SA), and jasmonic acid (JA). Major alterations have been detected in protein biosynthesis, energy metabolism, photosynthesis, protein folding, detoxification, and cell wall biosynthesis during the stress response of barley (Sicher et al., 2012; Rollins et al., 2013).

The wealth of knowledge gathered on barley genetics, genomics, diversity, genetic transformation, and stress responses makes this crop a platform for dissecting tolerance mechanism that can be then exploited in other crops, particularly cereals. Despite the complex nature of abiotic stress tolerance, single genes from barley can have potential in biotechnological crop improvement. In this minireview, our aim is to highlight notable genes from barley that may be used to improve plants for abiotic stress tolerance, with an emphasis on TFs.

### Overview of stress adaptation genes in barley

**Figure 1:**
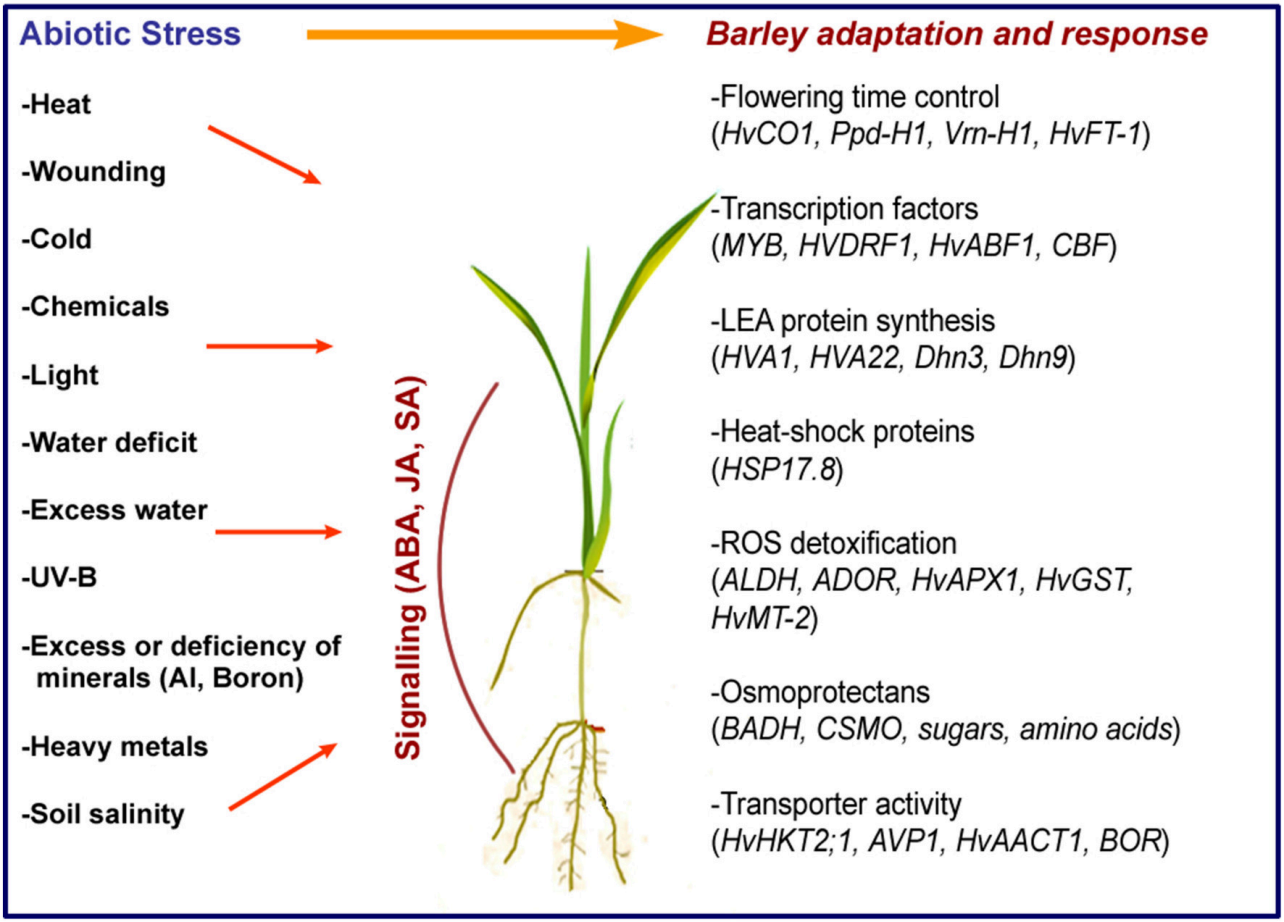
**Abiotic stress factors and main genes involved in adaptation and response in barley**.

Barley can cope with many abiotic stress factors, single or combined, and several genes involved have been identified (Figure 1). One of the factors behind the natural tolerance of barley to abiotic stresses is early flowering, which ensures that pollination, seed development, and maturation occur in an optimum time period. Major genes affecting flowering time in barley have been identified and shown to be mainly related with vernalization, photoperiod, and circadian clock (Turner et al., 2005; von Zitzewitz et al., 2011). For example, *HvCEN* and *HvLux1* control flowering time, while the circadian clock gene *Ppd-H1* regulates photoperiod-related output genes (Campoli et al., 2012; Comadran et al., 2012).

Expression of antioxidant enzymes such as *HvAPX1, HvMT-2*, and *HvGST1*, accumulation of osmolytes and synthesis of heat-shock proteins (e.g., HSP17.8) are among the initial responses to stress mainly generated by reactive oxygen species (ROS; Guo et al., 2009; Witzel et al., 2009). By the induction of transcription factors including *MYB, HvDRF1, HvDREB, HvABF1*, barley responds to stress with a large, partially elucidated network of genes. For instance, the expression of *HvCBF* genes at the Fr-H2 locus, which is controlled by a vernalisation TF gene, VRN-H1, affects frost tolerance (Stockinger et al., 2007).

A group of genes, *HVA1, HVA22, Dhn3*, and *Dhn9* encode proteins that bind to membrane phospholipids, ions and water, and protect cells by still unknown mechanisms. Overexpression of Na transporters (*HvHKT2;1*) were shown to contribute to the regulation of Na^+^/K^+^ homeostasis in barley during high salinity stress (Mian et al., 2011). In fact, K^+^ retention ability and limitation of Na^+^ uptake partially explains the tolerance of barley to ion toxicity and high salinity (Adem et al., 2014).

### Transcription factors: key players for stress tolerance

Engineering the regulatory machinery through transcription factors controlling the expression of stress-related genes is a promising approach to increase abiotic stress tolerance. Many transcription factors including DREB/CBF, ABF, AP2/ERF, bZIP, NAC, MYB, MYC, HD-ZIP, bHLH, NF-Y, EAR, and WRKY are known to be responsible for transcriptional reprogramming in response to abiotic stress conditions in plants. Several of these transcription factors have been cloned and characterized both at the genomic and protein levels in barley, and functionally proven to be useful for engineering stress tolerance in transgenic plants (Table 1).

**Table 1:** **Features of stress-related transcription factors (TFs) cloned and characterized from barley and their use in transgenic approaches**.

**Barley transcription factor**	**ABA-induction**	**Binding site (Cis-element)**	**Use for transgenics/promoter**	**Tolerance to**	**References**
*HvDRF1*	+	T(T/A)ACCGCCTT	No	Drought, salinity	Xue and Loveridge, 2004
*HvCBF4*	−	CRT/DRE1/DRE2	Yes/*Ubi*	Drought, salinity cold	Oh et al., 2007
*HvDREB1*	−	DRE/CTE	Yes/*CaMV 35S*	Salinity	Xu et al., 2009
*HvDREB1A*	nd	DRE/CTE	Yes/*CaMV 35S*	Drought, salinity	James et al., 2008
*HvRAF*	−	GCC-box, CRT/DRE	Yes/*CaMV 35S*	Salinity	Jung et al., 2007
*HvSNAC1*	+	–	Yes/*Ubi*	Drought	Al Abdallat et al., 2014
*HvWRKY38*	+	W-box[(T)(T)TGAC(C/T)]	Yes/*CaMV 35S*	Dehydration	Xiong et al., 2010

*nd, not determined*.

The effectiveness of a TF in regulating many genes at a time is determined by its affinity to specific DNA sequences and its binding capacity to the promoter. Most of the promoter binding sites of barley TFs have been characterized (Table 1), and subtle changes in these motifs recently appeared to be important in determination of the binding affinity of TFs (Singh and Laxmi, 2015). As the natural ability of barley in coping with many abiotic stress factors suggests better DNA-binding specificity of TFs in transcriptional regulation of stress responsive genes, we believe barley TFs can be considered as promising candidates to increase abiotic stress tolerance of other crops.

WRKY TFs are a very large family of zinc finger TFs known to regulate temporal and spatial expression of specific genes during development and in response to environmental stimuli such as wounding, pathogen infection, or abiotic stresses. WRKY TFs have been studied in detail in numerous plant species including barley (Li et al., 2014). Constitutive expression of *HvWRKY38* in bahiagrass (*Paspalum notatum* Flugge) caused better water retention capacity of transgenic plants during dehydration, and better recovery and rehydration with increased biomass production (Xiong et al., 2010).

DREB1/CBF and DREB2, induced by cold and dehydration in barley, respectively, belong to dehydration-responsive element binding protein/C-repeat binding factor family of TFs regulating expression of abiotic stress-related genes (Agarwal et al., 2006). An ortholog of the DREB1A TF isolated from a xeric, wild barley (*H. spontaneum* L.) under the transcriptional control of the stress-inducible *HVA1* promoter was shown to enhance survival and biomass production upon severe salt stress and repeated cycles of severe dehydration stress in bahiagrass (James et al., 2008). Similarly, the overexpression of the *HvDREB1* gene isolated from barley leaves increased salt stress tolerance in *Arabidopsis* (Xu et al., 2009).

The plant-specific NAC TFs are a major TF family with roles in regulation of several developmental programs and abiotic and biotic stress responsive genes (Nakashima et al., 2012; Puranik et al., 2012). The barley *HvNAC6* gene acting as a regulator of basal resistance against the biotrophic pathogen *Blumeria graminis* f. sp. *hordei*, was shown to mediate ABA-dependent defense responses in barley (Chen et al., 2013). Overexpression of the isoform *HvSNAC1* in barley increased drought tolerance (Al Abdallat et al., 2014), suggesting that this gene can be a tool for increasing barley productivity under drought conditions. A very recent study indicated the potential of overexpression of the same gene to enhance resistance of barley to *Ramularia* leaf spot (McGrann et al., 2015).

One of the earliest reports on isolation of a low-temperature induced AP2 domain and C-repeat/dehydration responsive element containing proteins identified HvCBF1 and HvCBF2, transcriptional activators of cold-responsive genes in barley (Xue, 2002, 2003). Indeed, CRF/DREBs mainly regulate freezing tolerance (Jeknić et al., 2014). Overexpression of *HvCBF4* in rice resulted in enhanced tolerance to drought, salt, and cold stresses at the seedling level (Oh et al., 2007).

HvRAF (barley root abundant factor), an ethylene response factor (ERF)-type TF, was shown to regulate transcriptional induction of various stress-responsive genes including PDF1.2, JR3, PR1, PR5, KIN2, and GSH1, and to confer higher seed germination and root growth with high salinity in transgenic *Arabidopsis*, in addition to enhanced resistance to *Ralstonia solanacearum* (Jung et al., 2007).

### Modifying transporter activity for stress tolerance

Plants have developed efficient strategies to maintain ion concentration in the cytoplasm at low levels. Transporters such as Na^+^/H^+^ and K^+^/H^+^ antiporters (NHXs), sucrose transporters and amino acid transporters have important roles to keep this balance. A group of transporters including NHXs, high affinity K^+^ transporters (HKTs), and salt overly sensitive 1 (SOS1) have been shown to maintain intracellular ion and pH homeostasis, and also contribute to the regulation of a wide variety of physiological processes associated with growth and development (Bassil et al., 2012).

Transgenic barley lines overexpressing a subfamily HKT transporter (*HvHKT2;1*) showed improved biomass production under salt stress (100 mM NaCl) probably through Na^+^ exclusion or accumulation of excessive Na^+^ in the leaves (Mian et al., 2011). The *HvNHX2* gene driven by the CaMV 35S promoter was introduced into two cultivars of potato, resulting in improved NaCl tolerance of one of the cultivars (Bayat et al., 2010). Bayat et al. (2011) also introduced *HvNHX2* in *Arabidopsis thaliana* and showed that transgenic plants grew normally at 200 mM NaCl.

In acid soils, aluminum (Al^3+^) can be toxic for plants. Over expression of the barley *HvAACT1* encoding a citrate transporter enhanced the Al^3+^ tolerance in barley and wheat (*Triticum aestivum*; Zhou et al., 2013). Besides, Fujii et al. (2012) showed that 1kb-insertion upstream of the coding region altered expression patterns of *HvAACT1*, leading to enhancement of Al^3+^ tolerance in barley cv. Morex.

Boron toxicity can severely limit crop production worldwide and is best combated by using tolerant varieties. Sutton et al. (2007) demonstrated that increased copy number of *Bot1* encoding a boron efflux transporter is the base of boron-toxicity tolerance in an African barley landrace containing four copies of the gene.

Iron deficiency is a major cause of reduced plant productivity in alkaline soils. Constitutive expression of a barley iron-phytosiderophore transporter (*HvYS1*) in transgenic rice increased iron uptake from alkaline soil (Gómez-Galera et al., 2012).

### A well-known success story: the *Hva1* gene for drought tolerance

LEA proteins are a well-known group of proteins characterized by hydrophilic nature, large size, and high accumulation during seed desiccation and in response to abiotic stresses (Bhatnagar-Mathur et al., 2008). HVA1, a 22kDA group 3 LEA protein expressed in the barley aleurone, is the first characterized and most studied barley LEA protein, having the potential to enhance abiotic stress tolerance through transgenic approaches.

*HVA1* expression increased drought tolerance of spring wheat, conferring higher biomass production and water use efficiency under greenhouse drought conditions (Sivamani et al., 2000). Bahieldin et al. (2005) reported improvement in drought tolerance in four independent T4 transgenic lines tested in nine field experiments over six growing seasons, and indicated that the field performance of lines was correlated with the level of *HVA1* transgene expression. Constitutive overexpression of *HVA1* in rice cv. Nipponbare increased tolerance to water deficit and salinity as shown by delayed damage symptoms and improved recovery (Xu et al., 1996). Further analysis of the T2 generation of these transgenic lines under prolonged drought stress indicated the possibility of better cell membrane protection with *HVA1* overexpression (Babu et al., 2004). Similarly, analysis of third generation transgenic rice plants (cv. Pusa Basmati 1) revealed improved cell integrity in transgenic plants under salt and drought stress conditions (Rohila et al., 2002). *HVA1* expression by an ABA/stress-inducible promoter resulted in improved root architecture and better tolerance to osmotic, salt, drought and cold stresses in transgenic rice (Chen et al., 2015). In a recent study, expression of *HVA1* in transgenic maize plants conferred survival under strong drought and tolerance to 100–300 mM NaCl in the T3 generation (Nguyen and Sticklen, 2013). The same group reported that co-expression of *HVA1* with E. coli mtlD (mannitol-1-phosphate dehydrogenase) in maize was more effective under drought stress, and capable to enhance shoot and root growth under salt stress when compared to transgenic plants expressing either gene, and underlined the potential of their co-expression for improvement of abiotic stress tolerance (Nguyen et al., 2013).

Transformation of *HVA1* into three oat (*Avena sativa* L.) cultivars resulted in better tolerance to osmotic (salt and mannitol) stresses compared to non-transgenic control plants (Maqbool et al., 2002). Constitutive or stress-inducible *HVA1* expression in drought-intolerant creeping bentgrass (*Agrostis stolonifera* var. *palustris*) resulted in higher turf quality and lower leaf wilting under water deficiency (Fu et al., 2007). Protection of stability of plasma and chloroplastic membranes under drought and salt stress conditions were observed in mulberry (*Morus indica*) transformed *HVA1* under the constitutive actin1 promoter (Lal et al., 2008). The same transgenic mulberry lines also showed better cold tolerance (Checker et al., 2012). Seedlings of common bean (*Phaseolus vulgaris* L) transgenic lines expressing *HVA1* under the control of the 35S promoter displayed enhanced drought tolerance and increased root length (Kwapata et al., 2012).

## Conclusions and future perspectives

Abiotic stress tolerant crops will probably be key for food security by helping agriculture to cope with climatic change (IPCC, 2015). Barley can provide a significant source of genes for stress tolerance due to its high diversity and adaptability.

Exploiting TFs in the design of stress tolerant transgenic plants has been proposed as a more effective tool than expressing single genes (Cominelli et al., 2013). In fact, overexpression of barley *HvWRKY38, HvDREB1, HvSNAC1*, and *HvCBF4* has proven to be very effective in conferring abiotic stress tolerance to other species, and provided tolerance to multiple stresses via both ABA-dependent and -independent pathways (Table 1). Complete functional analyses of barley TFs are still needed to understand regulatory networks related to abiotic stress responses and to reveal the cross-talk between different signaling pathways during stress adaptation.

Single gene transformation, however, can provide good results as indicated by the performance of wheat and rice plants expressing the barley *HVA1* LEA protein: in field conditions they have shown improved tolerance to salinity and drought. A less investigated group of genes from barley are membrane transporters that regulate ionic homeostasis in cells, and may have a high potential for creating cultivars with better tolerance to salinity and other mineral toxicities in various crops.

In conclusion, despite our knowledge toward resolving barley's high survival and adaptability in stressful environment is still limited, several stress tolerance genes have been characterized well enough to move them from basic research to implementation in crops. The wild relatives of barley can be of particular interest (Shavrukov et al., 2010; Uçarlı et al., 2016), by providing a range of allelic variants that could explain the degree of adaptive competence and plasticity of *Hordeum* and be used in plant breeding efforts for stress tolerance.

## Author contributions

FG has outlined the idea and wrote the manuscript with ZÖ and CU. DR contributed to critical reading and edition of the manuscript.

### Conflict of interest statement

The authors declare that the research was conducted in the absence of any commercial or financial relationships that could be construed as a potential conflict of interest.
